# Gp130-mediated STAT3 activation by *S*-propargyl-cysteine, an endogenous hydrogen sulfide initiator, prevents doxorubicin-induced cardiotoxicity

**DOI:** 10.1038/cddis.2016.209

**Published:** 2016-08-18

**Authors:** J Wu, W Guo, S-Z Lin, Z-J Wang, J-T Kan, S-Y Chen, Y-Z Zhu

**Affiliations:** 1 Shanghai Key Laboratory of Bioactive Small Molecules, Department of Pharmacology, School of Pharmacy, Fudan University, Shanghai, China; 2 Department of Cardiovascular Surgery, Guangdong General Hospital, Guangzhou, Guangdong, China; 3 School of Pharmacy, Macau University of Science & Technology, Macau, China

## Abstract

Doxorubicin (Dox) could trigger a large amount of apoptotic cells in the myocardium, which leads to dilated cardiomyopathy and heart failure. *S*-propargyl-cysteine (SPRC), a producing agent of endogenous hydrogen sulfide (H_2_S), possesses cardioprotective efficacy. However, the specific effect and mechanism of SPRC in Dox-induced cardiotoxicity remain elusive. Given gp130 with its main downstream signaling molecule, signal transducer and activator of transcription 3 (STAT3), is involved in cardiac myocyte survival and growth; the present study was performed to elucidate whether SPRC counteracts Dox-induced cardiotoxicity, and if so, whether the gp130/STAT3 pathway is involved in this cardioprotective activity. SPRC stimulated the activation of STAT3 via gp130-mediated transduction tunnel *in vitro* and *in vivo*. In Dox-stimulated cardiotoxicity, SPRC enhanced cell viability, restored expression of gp130/STAT3-regulated downstream genes, inhibited apoptosis and oxidative stress, and antagonized mitochondrial dysfunction and intracellular Ca^2+^ overload. Intriguingly, blockade of gp130/STAT3 signaling abrogated all these beneficial capacities of SPRC. Our findings present the first piece of evidence for the therapeutic properties of SPRC in alleviating Dox cardiotoxicity, which could be attributed to the activation of gp130-mediated STAT3 signaling. This will offer a novel molecular basis and therapeutic strategy of H_2_S donor for the treatment of heart failure.

Studies have proven that gp130-mediated signals transduce both cytoprotective and hypertrophic responses in the heart.^1^ The fundamental role for gp130-dependent pathway in heart failure has been elucidated using mice that harbor a ventricular-restricted knockout of the gp130.^2^ By contrast, activation of gp130 promotes cardiomyocyte survival by inhibiting apoptosis.^1^ Accordingly, identifying the downstream pathways by which gp130-dependent ligands can promote cardiac myocyte survival has become of critical interest. The signal transducer and activator of transcription 3 (STAT3) is an essential molecule downstream of gp130, which is activated under various stressful conditions, such as pressure-overload and myocardial infarction.^1^ For gp130-triggered STAT3 activation, gp130 is firstly phosphorylated by JAK, and the side chains of gp130 serve as docking sites for latent transcription factors of STAT3. STAT3 is activated when tyrosine 705 (Y705) is phosphorylated.^3^ Subsequently phosphorylated STAT3 molecules dimerize and translocate into the nucleus, where they bind to specific DNA response elements and induce the transcription of target genes, including antiapoptotic proteins (MCL-1, Bcl-2 and Bcl-X_L_)^4, 5, 6^ and proliferation regulatory proteins (survivin and cyclin D1).^4, 7, 8^ Gp130/STAT3 activation has both antiapoptotic and proliferative effects, and more importantly protects cardiomyocytes from ischemia/reperfusion or hypoxia/reoxygenation injury.^9, 10, 11, 12^ Thus, targeting gp130/STAT3 signaling may be a promising strategy for therapeutic intervention of heart failure.

Doxorubicin (Dox), an anthracycline derivative, is effective for a broad range of soft and solid human malignancies.^13^ However, its clinical use is challenged by the risk of serious cardiotoxicity, which causes dilated cardiac dysfunction and congestive heart failure associated with the development of irreversible cardiomyopathy.^14^ Given the number of patients at risk of Dox-induced cardiotoxicity is increasing,^15^ urgent needs to prevent severe morbidity and possible mortality in humans prevail. The molecular mechanism involved in Dox-induced cardiotoxicity has been proposed to account for increasing reactive oxygen species (ROS) production, caspase activation, altered calcium handling and mitochondrial injury.^16, 17^ It was demonstrated that transgenic mice with cardiac-specific overexpression of *STAT3* gene are protected against Dox-induced cardiomyopathy.^18^ Therefore, understanding how STAT3 activation can be modulated would provide new opportunities to develop effective therapeutics for Dox cardiotoxicity.

Preclinical studies investigating cardiovascular diseases have clarified that the administration of physiological or pharmacological levels of hydrogen sulfide (H_2_S) alleviates myocardial injury, protects blood vessels, limits inflammation and regulates blood pressure.^19^ *S*-propargyl-cysteine (SPRC, also reported as ZYZ-802), designed and synthesized by our group, is a novel producing compound of endogenous H_2_S. SPRC promotes the activity of cystathionine-*γ*-lyase (CSE, a metabolic enzyme producing endogenous H_2_S) and then augments H_2_S level in mammalian plasma and tissues.^20^ Our previous studies confirmed that SPRC exerts extensive protective effects on cardiovascular diseases via anti-oxidative, anti-inflammatory, and proangiogenic mechanisms.^20, 21, 22, 23^ However, the definite role and mechanism of SPRC in Dox-induced cardiotoxicity are not well established. Herein, the protective effects of SPRC against Dox-induced cardiotoxicity were explored in detail. Because gp130-mediated STAT3 activation plays a fundamental role in cardioprotection, the modulation of gp130/STAT3 signaling by SPRC was delineated in both cultured cardiomyocytes and mouse hearts.

## Results

### Activation of gp130-initiated STAT3 by SPRC in cardiomyocytes

**Figure 1:**
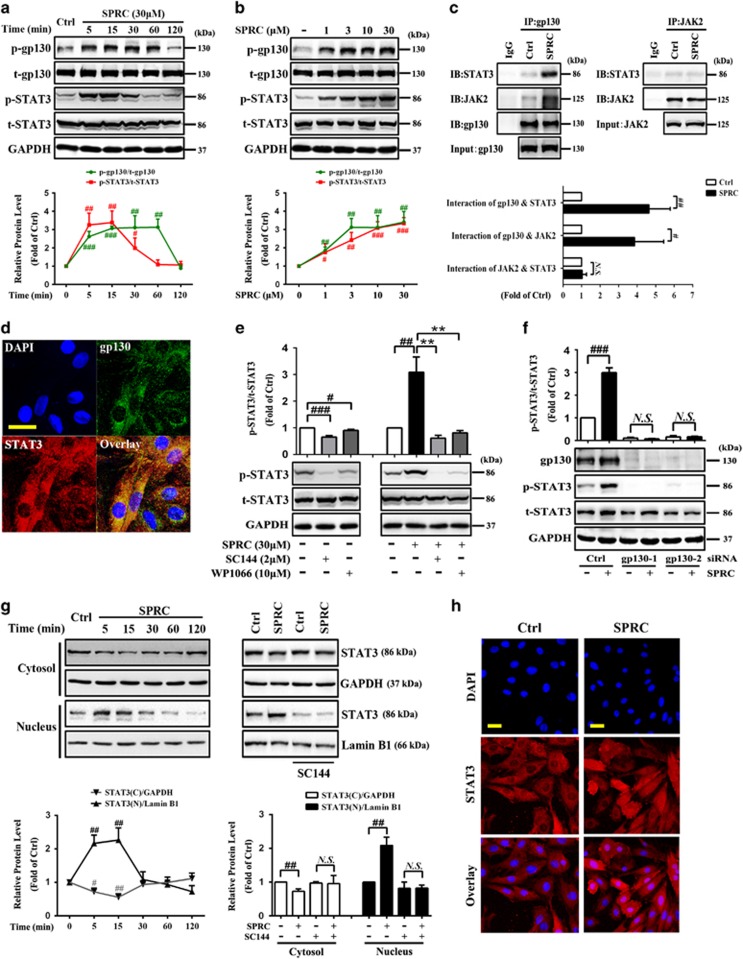
SPRC induces STAT3 phosphorylation and nuclear translocation via gp130 in cardiomyocytes. H9c2 cardiac myocytes were treated either (**a**) with 30 *μ*M SPRC for the indicated durations or (**b**) with increasing concentrations of SPRC for 10 min. Cell lysates were collected and subjected to western blot for evaluating phospho-gp130 and phospho-STAT3. (**c**) H9c2 cells were treated with SPRC (30 *μ*M) for 10 min. Cells lysates were immunoprecipitated with gp130 or JAK2 antibody, and the bound proteins were fractionated on SDS-PAGE gel, then analyzed by western blot with antibodies indicated. (**d**) Cells were incubated with SPRC (30 *μ*M) for 10 min, and then double-immunostained for gp130 and STAT3 and the nuclei were visualized by DAPI staining. Scale bar, 50 *μ*m. (**e**) Cells were pretreated with 2 *μ*M SC144 (gp130 inhibitor) or 10 *μ*M WP1066 (STAT3 inhibitor) for 1 h, followed by SPRC stimulation. (**f**) Cells were incubated with SPRC after gp130 silencing, which was conducted by siRNA transfection for 48 h. (**g**) Cells were treated with SPRC for the indicated durations, or pretreated with/without SC144 for 1 h before SPRC stimulation for 10 min. Levels of STAT3 in the cytosol and nucleus were analyzed by western blot. GAPDH and Lamin B1 were used as loading control for cytosolic and nuclear proteins, respectively. (**h**) STAT3 nuclear translocation after 10 min of SPRC treatment was detected by confocal microscopy. Scale bars, 50 *μ*m. Values are presented as mean±S.D. from *n*=3 replicates. ^#^*P*<0.05, ^##^*P*<0.01, ^###^*P*<0.001 compared with the control group; ^**^P<0.01 compared with the SPRC-treated group. NS, nonsignificant (*P*>0.05)

The gp130 receptor system and its main downstream mediator, STAT3, play a key role in cardioprotection.^24^ As shown in Figures 1a and b, SPRC treatment transiently increased the phosphorylation of gp130 (S782) and STAT3 (Y705) in cardiomyocytes in time- and dose-dependent manner (*P*<0.05). It has been established that the activated JAK1 or JAK2 phosphorylate gp130, which then serves as the docking receptor for STAT3.^25^ To investigate whether SPRC affects the recruitment of STAT3 to gp130, we performed co-immunoprecipitation (Co-IP) assay. After SPRC stimulation, the interaction of gp130-STAT3 and gp130-JAK2 was markedly enhanced (*P*<0.05), whereas STAT3 acted negatively with JAK2, indicating that SPRC was able to directly stimulate binding of STAT3 to gp130 receptor and thus possibly transmit a gp130-initiated signal (Figure 1c). In addition, double fluorescent labeling experiment exhibited that STAT3 was co-localized with gp130 after SPRC treatment (Figure 1d). The STAT3 activation by SPRC was blunted by SC144 (*P*<0.01; Figure 1e), which could bind to gp130, induce gp130 phosphorylation and deglycosylation, and eventually abrogate STAT3 phosphorylation and nuclear translocation.^8^ Consistent result was observed after transfecting cells with gp130 small interfering RNAs (siRNAs) to knock down its expression (Figure 1f). As displayed in Figure 1g, 5–15 min of SPRC stimulation dramatically decreased the cytosolic STAT3 level but elevated its translocation into the nucleus, which could be abolished by SC144 (*P*<0.05). Direct observation of STAT3 localization after SPRC treatment was obtained using confocal microscopy. Augmentation of STAT3 in the nucleus was visualized in SPRC-stimulated cells in contrast to that in the control group (Figure 1h).

### Enhancement of gp130/STAT3 activity by SPRC in Dox-induced cardiomyocytes

**Figure 2:**
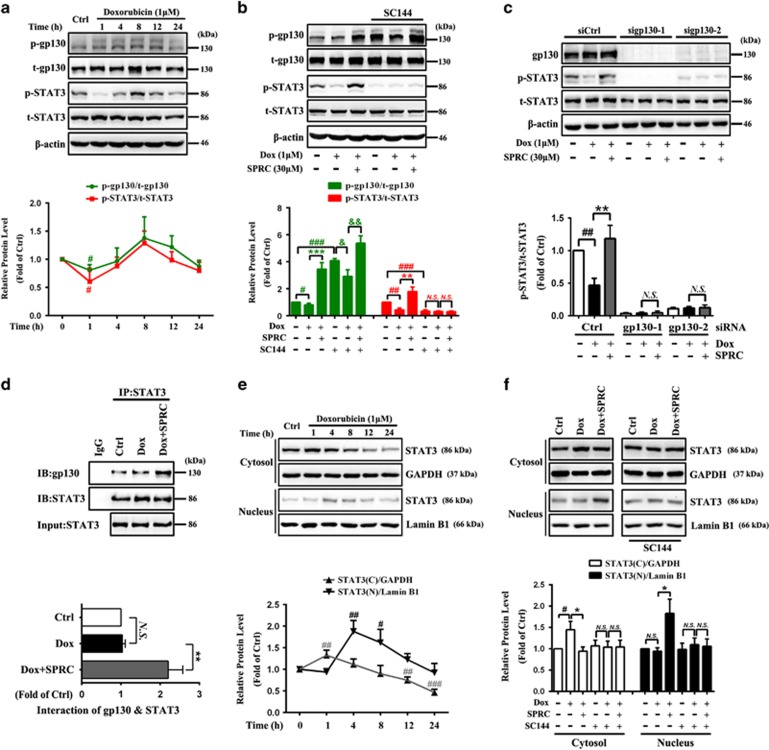
SPRC enhances STfAT3 activity through a gp130-mediated mechanism in Dox-induced cardiomyocytes. (**a**) H9c2 cells were incubated with Dox (1 *μ*M) during different time periods (1, 4, 8, 12 and 24 h). Phosphorylation of gp130 and STAT3 was determined by western blot. (**b**) One hour after incubation of SC144 (2 *μ*M) and (**c**) 48 h after transfection with control siRNA or gp130 siRNAs, cardiomyocytes were treated with SPRC for 30 min prior to stimulation with Dox during 1 h. (**d**) Cells were pretreated with SPRC (30 *μ*M) for 30 min, and then stimulated with Dox for 1 h. The interaction between STAT3 and gp130 was determined by co-immunoprecipitation analysis. (**e**) Cells were stimulated with Dox for indicated time periods and (**f**) pretreated by SC144 (2 *μ*M, 1 h), and then SPRC (30 *μ*M, 30 min) following the stimulation of Dox during 1 h. STAT3 expression in the cytosolic and nuclear fractions was detected. Values are presented as mean±S.D. from *n*=3 replicates. ^#^*P*<0.05, ^##^*P*<0.01, ^###^*P*<0.001 compared with the control group; ^*^P<0.05, ^**^P<0.01, ^***^P<0.001 compared with the Dox group; ^&^*P*<0.05, ^&&^*P*<0.01 compared with the SC144+Dox group. NS, nonsignificant (*P*>0.05)

To confirm the facilitating effect of SPRC on gp130/STAT3 activation, a Dox-induced cardiac injury model was established *in vitro*. As shown in Figure 2a, a substantial decrease in the phospho-STAT3 level was detected 1 h after Dox treatment (*P*<0.05). By comparison, such reduction was reversed by SPRC (*P*<0.01, Figure 2b). Similar to non-Dox-stimulated cells, pretreatment with SC144 depleted SPRC-induced STAT3 phosphorylation (Figure 2b). Likewise, gp130 siRNAs blocked the elevated STAT3 phosphorylation by SPRC(Figure 2c). Additionally, SPRC markedly increased the binding of STAT3 to gp130 in Dox-induced cardiomyocytes, examined by reciprocal immunoprecipitation against endogenous STAT3 (*P*<0.01; Figure 2d). All these findings demonstrated that SPRC probably targeted gp130 and triggered the signal transduction from gp130 to STAT3. Upon 1 h of Dox stimulation, no apparent altered STAT3 nuclear translocation was detected compared with control cells (Figure 2e), whereas SPRC obviously increased the STAT3 level in the nuclear fraction, which could be suppressed by SC144 (*P*<0.05; Figure 2f).

### Protective effect of SPRC against Dox-induced cell injury is mediated by gp130/STAT3 signaling

**Figure 3:**
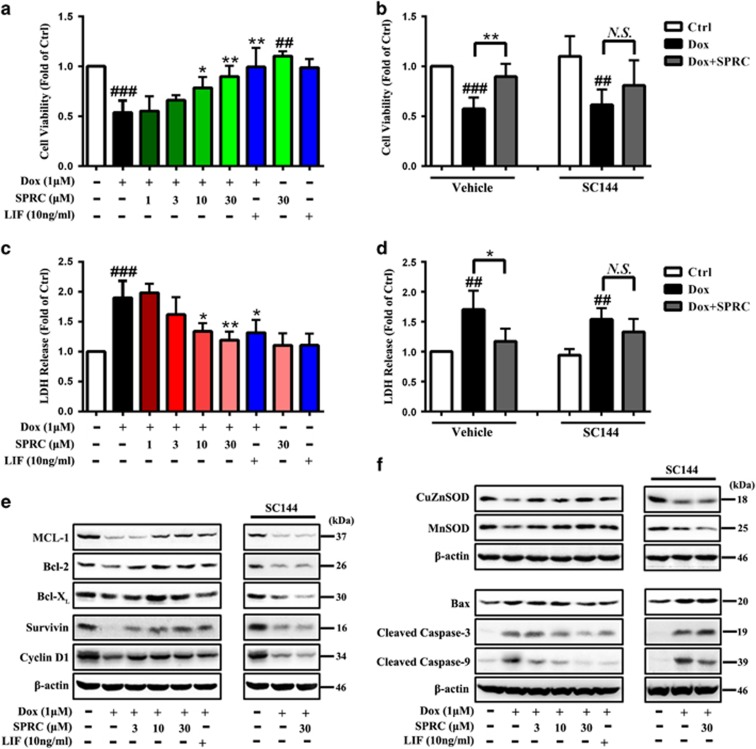
SPRC attenuates Dox-induced cardiomyocyte death via gp130/STAT3 signaling. H9c2 cells were incubated with SPRC (30 *μ*M), LIF (gp130/STAT3 pathway activator, 10 ng/ml) for 24 h or pretreated with/without SC144 (2 *μ*M, 1 h) before indicated concentrations of SPRC or LIF treatment (30 min), followed by Dox stimulation for 24 h. (**a** and**b**) Cell viability and (**c** and**d**) LDH release was evaluated using CCK-8 assay and LDH assay. (**e**and**f**) gp130/STAT3-associated genes' expression in cardiomyocytes was measured by western blot analysis. Values are presented as mean±S.D. from *n*=6 replicates. ^##^*P*<0.01, ^###^*P*<0.001 compared with the control group; ^*^P<0.05, ^**^P<0.01 compared with the Dox group. NS, nonsignificant (*P*>0.05)

The possible modulation of gp130/STAT3 signaling by SPRC in Dox-induced cell death was next evaluated. Leukemia inhibitory factor (LIF), an IL-6-related cytokine that binds the gp130 receptor chain and subsequently activates STAT3, has been reported to reduce myocyte death after Dox treatment.^26^ Therefore, LIF was utilized as a reference drug in comparison with SPRC. The cardiomyocytes were exposed to Dox for 24 h and cell survival percentages were measured using CCK-8 and lactate dehydrogenase (LDH) release assay. As illustrated in Figures 3a and c, cells receiving SPRC (30 *μ*M) or LIF (10 ng/ml) treatment for 24 h showed no significant increases in cell death, indicating that SPRC at the concentrations used in this study was not cytotoxic. SPRC restored Dox-induced decrease in cell viability and increase in LDH leakage in a concentration-dependent manner (*P*<0.05; Figures 3a and c). These cell survival effects of SPRC could be significantly abolished by gp130/STAT3 signaling inhibitor, SC144 (Figures 3b and d).

A growing body of cancer research evidence has indicated that activation of gp130/STAT3 induces the expression of multiple survival, proliferation and antiapoptosis associated genes, such as *MCL-1*, *Bcl-2*, *Bcl-X_L_*, *Survivin* and *cyclin D1*.^4, 6, 7, 8^ Moreover, STAT3 downregulates proapoptotic genes including Bax and caspase enzymes.^27, 28^ In the cardiovascular system, the protective roles of STAT3 have been linked to a direct transcriptional upregulation of antioxidant enzymes such as MnSOD, as well as to the induction of antiapoptotic and cardioprotective proteins like Bcl-X_L_,^11, 29, 30^ demonstrating the critical importance of STAT3 activation in cardiomyocytes for survival and growth. As exhibited in Figures 3e and f, Dox caused a marked reduction in the expression of MCL-1, Bcl-2, Bcl-X_L_, Survivin, cyclin D1, CuZnSOD and MnSOD compared with vehicle control (*P*<0.01; Supplementary Figure S1). Meanwhile, obviously enhanced Bax, cleaved caspase-3 and cleaved caspase-9 expression was observed after Dox treatment (*P*<0.01; Figure 3f and Supplementary Figure S1). SPRC and LIF significantly antagonized these effects of Dox, and SC144 reversed the effects of SPRC (Figures 3e and f and Supplementary Figure S1).

### Inhibitory effect of SPRC on Dox-induced cardiomyocyte apoptosis is mediated by gp130/STAT3 signaling

**Figure 4:**
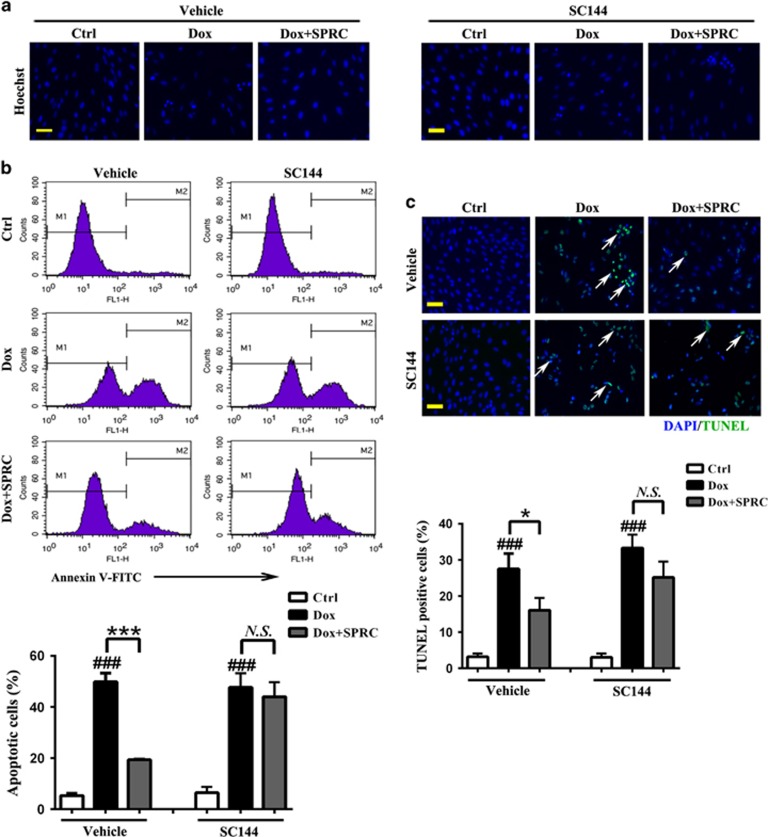
Suppression of gp130/STAT3 blocks the protective effect of SPRC against Dox-induced cell apoptosis. Cardiomyocytes were pretreated with/without SC144 before SPRC incubation, followed by Dox (1 *μ*M) stimulation for 24 h. (**a**) Morphological apoptosis was determined by Hoechst 33258 staining. Scale bars, 50 *μ*m. (**b**) Flow cytometry detection of apoptosis with Annexin V-FITC staining. (**c**) Immunofluorescent staining detection of apoptotic nuclei using TUNEL assay. Scale bars, 50 *μ*m. Values are presented as mean±S.D. from *n*=3 replicates. ^###^*P*<0.001 compared with the control group; ^*^P<0.05, ^***^P<0.001 compared with the Dox group. NS, nonsignificant (*P*>0.05)

The antiapoptotic effects of SPRC were analyzed using the Hoechst 33258, Annexin V-FITC and TUNEL staining. As shown in Figure 4a, normal cells were observed as round-shaped nuclei with homogeneous fluorescence intensity. Dox induced rapid broken nuclei of cells with heterogeneous intensity and chromatin condensation. Conversely, the SPRC-treated cells exhibited slight DNA condensation with a few fragmentations of chromatin. Figure 4b shows that Dox induced 49.8±3.5% apoptosis (Annexin V-positive staining cells), detected by FACS analysis. SPRC drastically lowered Dox-induced proportion of apoptotic cells to 19.3±0.3%. Furthermore, DNA fragmentation, a characteristic finding of apoptotic cells, was visualized by TUNEL staining. Consistently, distinct increases in TUNEL-positive nuclei were seen after Dox stimulation, but SPRC strongly reduced TUNEL-positive staining (*P*<0.05; Figure 4c and Supplementary Figure S2). All these antiapoptotic effects of SPRC were abrogated by SC144 (Figures 4a–c).

### Alleviation of Dox-induced ROS generation and mitochondrial dysfunction by SPRC is mediated by gp130/STAT3 signaling *in vitro*

**Figure 5:**
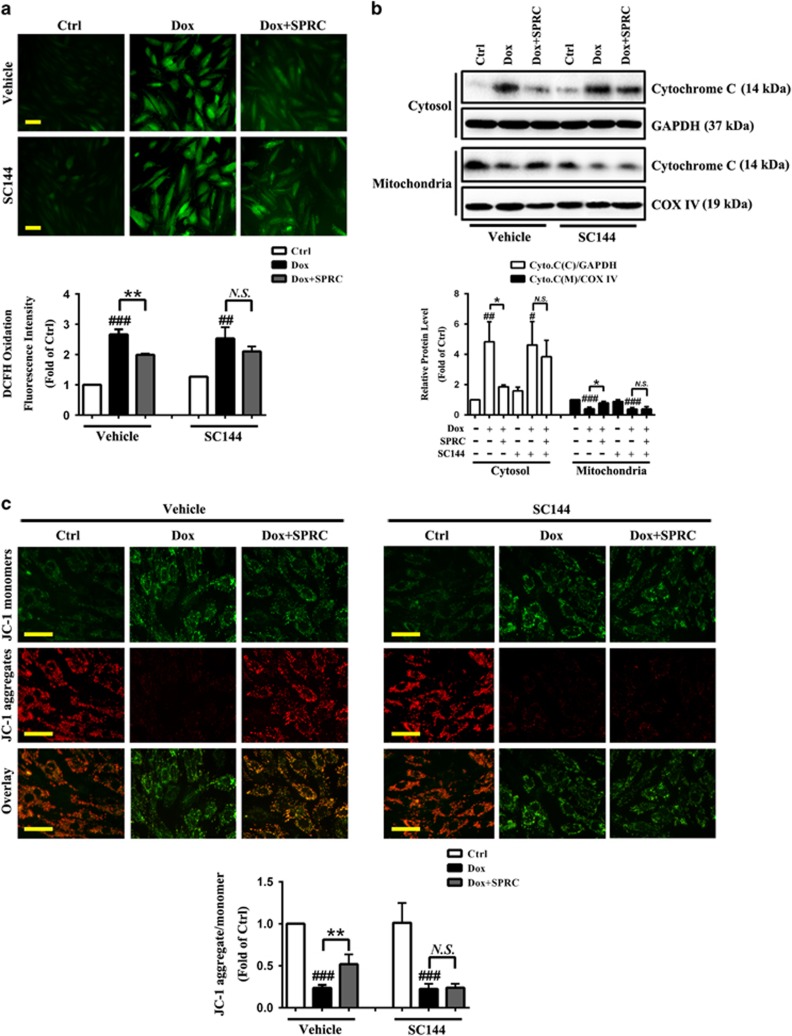
Suppression of gp130/STAT3 abolishes the inhibitory effect of SPRC on Dox-induced ROS generation and mitochondrial dysfunction. Cardiomyocytes were pretreated with/without SC144 before SPRC incubation, followed by Dox (1 *μ*M) stimulation for 24 h. (**a**) Staining of intracellular ROS by use of DCFH-DA and column bar graph of mean fluorescence intensity of DCF. Scale bars, 50 *μ*m. (**b**) Western blot analysis of cytochrome *c* release from mitochondria to cytosol. (**c**) Representative images and quantitative analysis of JC-1 fluorescence staining for the changes in mitochondrial membrane potential (*ΔΨm*). Scale bars, 50 *μ*m. Values are presented as mean±S.D. from *n*=3 replicates. ^#^*P*<0.05, ^##^*P*<0.01, ^###^*P*<0.001 compared with the control group; ^*^P<0.05, ^**^P<0.01 compared with the Dox group. NS, nonsignificant (*P*>0.05)

Principally, cellular ROS production is involved in Dox-induced cardiotoxicity.^31^ As shown in Figure 5a, Dox caused an approximately 2.7-fold increase in intracellular ROS generation as monitored by DCF fluorescence, which was significantly decreased by SPRC (*P*<0.01). Moreover, consistent with recent studies,^31, 32^ Dox-treated cells revealed the release of proapoptotic mitochondrial protein cytochrome *c* into cytosol. In contrast, SPRC prevented Dox-induced cytochrome *c* release (*P*<0.05; Figure 5b). Furthermore, mitochondrial membrane potential (*ΔΨm*) is one of key events during apoptosis. Mitochondrial permeability transition has been implicated in the collapse of *ΔΨm*.^33^ As displayed in Figure 5c, the vehicle control cells mostly exhibited brightly stained mitochondria emitting red fluorescence (derived from aggregates), whereas the Dox-stimulated cells produced green fluorescence (derived from monomers) indicative of mitochondrial depolarization and the collapse of *ΔΨm*, which was approximately 76% loss compared with the vehicle control cells, monitored by use of JC-1 dye. Conversely, SPRC-treated cells showed a significant preservation of red fluorescence in contrast with the Dox group (*P*<0.01). Importantly, the attenuation of Dox-induced ROS generation and mitochondrial dysfunction by SPRC was abolished by inhibiting gp130/STAT3 signaling (Figures 5a–c).

### Amelioration of Dox-induced [Ca^2+^]_i_ overload by SPRC is mediated by gp130/STAT3 signaling *in vitro*

**Figure 6:**
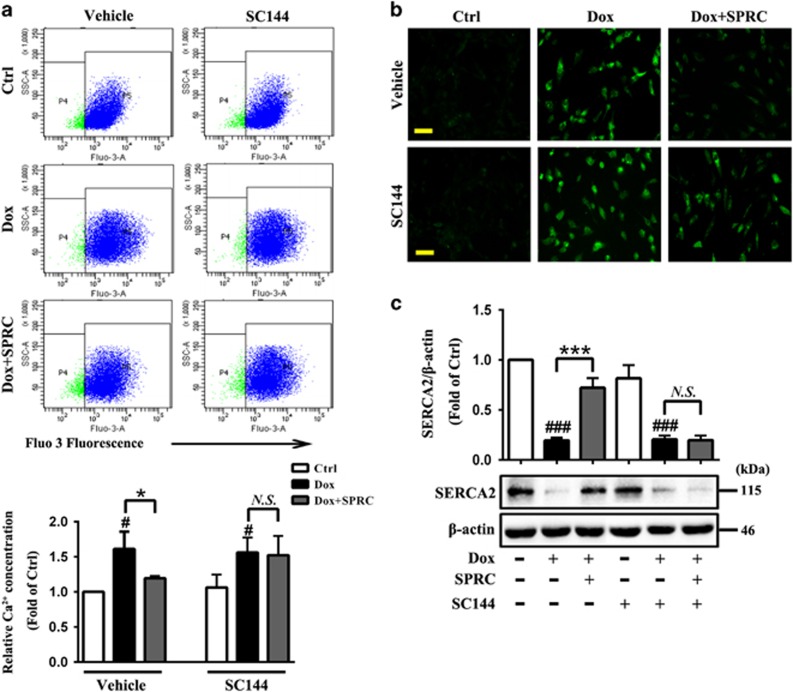
Suppression of gp130/STAT3 reduces the inhibitory effect of SPRC on Dox-induced [Ca^2+^]_i_ overload. Cardiomyocytes were pretreated with/without SC144 before SPRC incubation, followed by Dox (1 *μ*M) stimulation for 24 h. The intracellular [Ca^2+^]_i_ concentration was (**a**) quantified by flow cytometry and (**b**) visualized by fluorescence microscopy using fluo-3/AM probe, a fluorescent Ca^2+^-indicator dye. Scale bars, 50 *μ*m. (**c**) Representative western blot analysis of SERCA2. Values are presented as mean±S.D. from *n*=3 replicates. ^#^*P*<0.05, ^###^*P*<0.001 compared with the control group; ^*^P<0.05, ^***^P<0.001 compared with the Dox group. NS, nonsignificant (*P*>0.05)

Dox-mediated alternation of Ca^2+^ homeostasis is another possible mechanism of cardiotoxicity.^34^ As shown in Figures 6a and b, normal cells exhibited a low basal level of [Ca^2+^]_i_, but a 1.6-fold augmentation in intracellular [Ca^2+^]_i_ concentration was detected after Dox treatment. Nevertheless, SPRC suppressed Dox-induced [Ca^2+^]_i_ accumulation (*P*<0.05). To further determine whether SPRC protects against the adverse effects of Dox on the contractions of cardiomyocytes, the expression of sarcoplasmic/endoplasmic reticulum Ca^2+^ ATPase (SERCA2), an intracellular Ca^2+^ handing gene which indicates the changes in cardiomyocyte contractility, was assessed. SPRC treatment notably restored Dox-induced decrease in SERCA2 expression (*P*<0.001; Figure 6c). As expected, blocking of gp130/STAT3 signaling by SC144 significantly blunted these effects of SPRC (Figures 6a–c).

### Attenuation of Dox-induced heart injury by SPRC depends on gp130/STAT3 signaling

**Figure 7:**
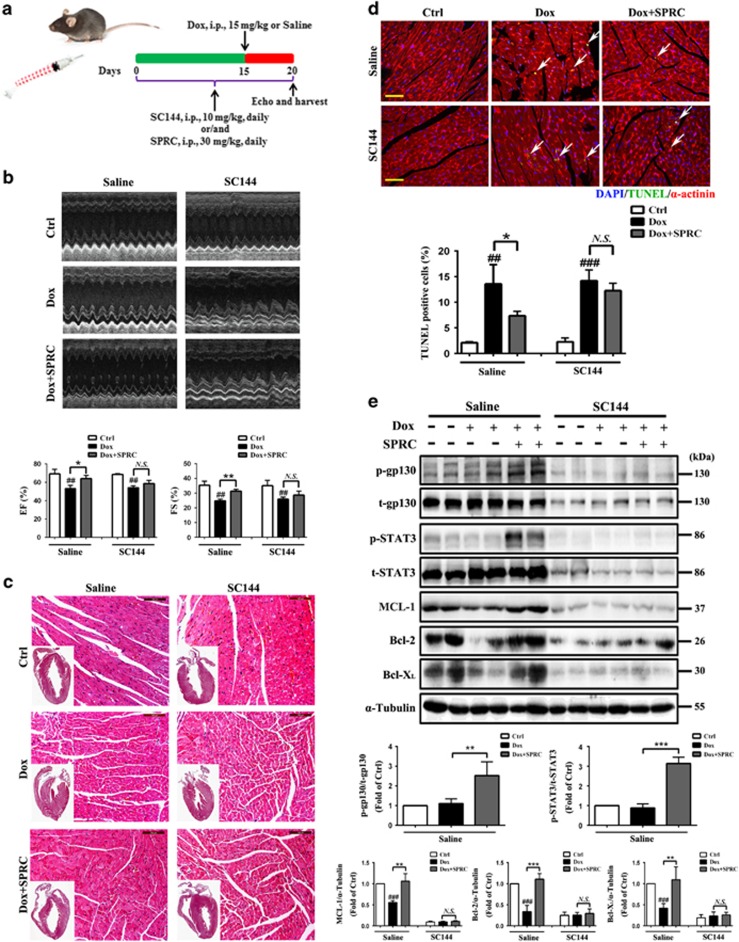
SPRC prevented Dox-induced heart injury though gp130/STAT3 signaling *in vivo*. (**a**) Experimental paradigm. C57BL/6 mice were administrated with SC144 (10 mg/kg/day) or/and SPRC (30 mg/kg/day) 2 weeks before Dox and 5 days after Dox stimulation (15 mg/kg), *n*=6 per group. (**b**) Representative echocardiographic graphs and quantification of ejection fraction (EF %) and fractional shortening (FS %). (**c**) Pathology changes were detected by H&E staining. Scale bars, 100 *μ*m. (**d**) DNA fragmentation of apoptotic cardiomyocytes in the myocardium was detected by TUNEL staining (green). Nuclei and cardiomyocytes were counterstained with DAPI (blue) and *α*-actinin (red). Scale bars, 100 *μ*m. (**e**) Protein levels of p-gp130, t-gp130, p-STAT3, t-STAT3, MCL-1, Bcl-2 and Bcl-X_L_ were analyzed by western blot. Values are presented as mean±S.D. from *n*=4 replicates. ^##^*P*<0.01, ^###^*P*<0.001 compared with the control group; ^*^P<0.05, ^**^P<0.01, ^***^P<0.001 compared with the Dox group. NS, nonsignificant (*P*>0.05)

To determine *in vivo* efficacy of SPRC, we tested its effect on an established heart failure model using Dox-treated mice. The mice were intraperitoneally administrated with SC144 or/and SPRC 14 days before Dox and 5 days after Dox, and then killed on day 20 (Figure 7a). Echocardiographic examination revealed that compared with saline treatment, Dox led to a significant loss of systolic function with decreased left ventricular ejection fraction (EF) and fractional shortening (FS), which was partially reversed by SPRC (*P*<0.05; Figure 7b). In hematoxylin and eosin (H&E)-stained myocardial samples, Dox-induced heart failure was associated with increased thickening of the left ventricular wall and decreased ventricular dilatation. Moreover, disarray of myofilament arrangement and focal tissue lysis became visible following Dox treatment. These pathological changes were alleviated by SPRC (Figure 7c). To evaluate the role of SPRC in Dox-induced cardiomyocyte apoptosis *in vivo*, the apoptotic cells in myocardial sections were identified by TUNEL assay. Consistent with our *in vitro* data, SPRC reduced the Dox-triggered apoptosis in heart (*P*<0.05; Figure 7d and Supplementary Figure S3). As shown in Figure 7e, SPRC elevated the levels of p-gp130, p-STAT3, MCL-1, Bcl-2 and Bcl-X_L_ in Dox-treated heart (*P*<0.01). The effects of SPRC on Dox-induced left ventricular dysfunction, myocardial injury and apoptosis, and increase in gp130/STAT3 effectors expression were all inhibited by SC144 treatment (Figures 7b–e), indicating that SPRC exerted heart protection through upregulation of gp130/STAT3 pathway.

## Discussion

Gp130 receptor-mediated signaling promotes cardiomyocyte survival, induces hypertrophy, and modulates cardiac extracellular matrix and cardiac function.^35^ Cardiac-specific disruption of gp130 results in increased apoptosis of cardiomyocytes in response to ischemia or mechanical stress.^1, 2^ Inactivation of STAT3 resulting from the loss of gp130 is a crucial event in heart failure.^35^ In this regard, the gp130/STAT3 signaling pathway plays a key functional role for cardiac adaption and protection in response to various forms of stress leading to heart failure. Our salient results revealed for the first time that SPRC mediates the protective function of gp130/STAT3 in response to Dox-induced cardiomyopathy in both cardiomyocytes and hearts.

Although application of Dox represents a powerful therapeutic option for various types of cancer treatment, its clinical utility is restricted by severe side effects, in particular cardiotoxicity.^14^ Thus, a better understanding of the mechanisms underlying Dox-induced cardiomyopathy will enable development of therapies. H_2_S, as a gaseous mediator, fulfills a wide range of physiological and pathological functions in the cardiovascular system.^19^ Recent studies have shown that exogenous H_2_S (NaHS) contributes to the protective effects in Dox cardiotoxicity partly by inhibition of ERK 1/2, p38 pathway and endoplasmic reticulum (ER) stress,^36, 37, 38^ demonstrating that H_2_S may offer a new therapeutic approach for Dox-induced cardiotoxicity.

Among possible mechanisms underlying the cardiotoxic effect of Dox that have been reported by numerous investigators, impaired STAT3 signaling appears to be essentially involved in the etiology of Dox-induced cardiomyopathy.^18^ This has been concluded by the finding that STAT3 mRNA expression in the heart is selectively and dramatically reduced by Dox treatment^39^ and by observations in mice with cardiac-specific deletion of STAT3 that are more susceptible to Dox-induced cardiac injury and development of heart failure.^40^ Oppositely, transgenic mice with cardiac-specific overexpression of STAT3 (STAT3-TG) displayed significantly increased survival rate compared with wild-type littermates.^18^ The STAT3-induced myocardial protection is likely achieved by promoting the expression of genes critical for the structural integrity and induction of cytoprotective factors. For example, the expression of ANF and CT-1 mRNA is enhanced in STAT3-TG subjected to Dox triggering cell growth and survival, thus preventing cardiac decompensation induced by Dox.^18^

Our previous study has proven that the proangiogenic effect of the endogenous H_2_S donor, SPRC, is mediated by STAT3 in spite of no direct binding of SPRC to STAT3 by the method of co-crystallization. SPRC could enhance the interaction between VEGFR2 and STAT3 as well as promote STAT3 nuclear translocation and its transcriptional activation of downstream promoters, particularly the *Vegf* promoter in human umbilical vein endothelial cells.^23^ Intriguingly, our present investigation elucidated that SPRC rapidly elevated STAT3 phosphorylation and nuclear translocation through a gp130-mediated mechanism in normal H9c2 cardiomyocytes (Figure 1), which further shed light on the beneficial role of SPRC in STAT3-related cardiovascular diseases. Our data further suggested that SPRC promoted gp130-STAT3 interaction with corresponding STAT3 activation *in vitro* (Figure 2), accompanied by the upregulation of gp130/STAT3-regulated cytoprotective and antiapoptotic proteins (e.g., MCL-1, Bcl-2 and Bcl-X_L_) expression following Dox treatment *in vitro* and *in vivo* (Figures 3 and 7). Such effect of SPRC is similar to the gp130 receptor-signaling cytokine, LIF, which has been reported to act as a survival factor to ameliorate myocardial cell damage.^11, 26, 41^ Additionally, SPRC was found to counteract Dox-induced Bax, Caspase-3 and -9 activation, and this capacity could also be abrogated by gp130/STAT3 signaling blockade (Figure 3). Although whether the transcriptional modulation of these proapoptotic molecules is involved in the STAT3-dependent mechanisms remains to be addressed, on the basis of our *in vitro* and *in vivo* results confirming that inhibition of gp130/STAT3 signaling reduced SPRC potency in cardioprotection, we conclude that SPRC-mediated cellular signals triggered by the gp130/STAT3 axis play a fundamental role in attenuating myocardial injury in response to Dox.

Increasing evidence has suggested that increased oxidative stress associated with an impaired antioxidant defense status plays a crucial role in Dox-induced subcellular remodeling, Ca^2+^-handling abnormalities and altered mitochondrial function, eventually culminating into cardiac dysfunction, with subsequent cardiomyopathy and heart failure.^15, 42, 43^ Importantly, excessive ROS production serves as an upstream trigger of the apoptosis cascade and ROS-induced apoptosis is a final common pathway for progressive heart failure.^44^ On the one hand, ROS generation correlates with intracellular Ca^2+^ accumulation, and interference with electron transport by ROS and intracellular Ca^2+^ results in the collapse of *ΔΨm* which is regarded as an irreversible point in death cascade.^45^ The release of cytochrome *c* from mitochondria to cytosol, and dissipation of *ΔΨm*, is linked to Dox-mediated apoptotic signaling and mitochondrial dysfunction.^31^ On the other hand, oxidative stress through cumulative Ca^2+^ and mitochondrial dysfunction causes depletion in ATP necessary in the process of myocardial fiber contractions.^46, 47^ It can also be a basis for pathological changes in Ca^2+^ regulation through SERCA2.^48, 49^ As a result, oxidative stress can lead to myocardial apoptosis, remodeling and contractility disturbances. In the present study, SPRC prevents myocardial apoptosis (reducing the number of apoptotic cells and generation of ROS, ameliorating alternation of Ca^2+^ homeostasis and disorder of mitochondrial function) induced by Dox and this protection required the activation of the pro-survival gp130/STAT3 pathway (Figures 4, 5, 6). Therefore, our findings unveils a novel mechanism and therapeutic strategy whereby activation of gp130/STAT3 signaling by SPRC mitigates the Dox-induced cell apoptosis, ROS formation, mitochondrial dysfunction and Ca^2+^ accumulation that are major causes of Dox-induced cardiotoxicity.

**Figure 8:**
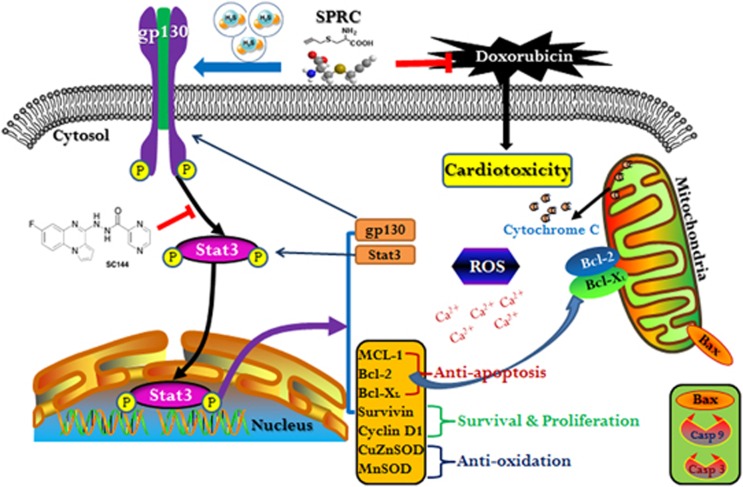
Schematic illustration of proposed mechanism of gp130/STAT3 signaling modulation by SPRC. SPRC activates STAT3 though gp130 in Dox-induced cardiomyocytes and hearts. SPRC attenuates Dox-induced cardiotoxicity via a mechanism involving the promotion of gp130-mediated STAT3 activity, leading to activation of STAT3-regulated cardioprotective molecules expression (e.g., MCL-1, Bcl-2, Bcl-X_L_, Survivin and MnSOD), mitigation of mitochondrial dysfunction, and suppression of ROS generation and [Ca^2+^]_i_ accumulation

In summary, although more distinct gp130/STAT3-dependent mechanisms still remain elusive, based on our studies, the cardioprotective effects of SPRC are possibly accounted for gp130/STAT3-mediated protection against Dox-induced cardiomyopathy (Figure 8). Clinical implementation of this promising candidate drug may help reducing anthracycline-related cardiotoxicity and improving the long-term outcome of antineoplastic treatment with anthracyclines.

## Materials and Methods

### Drugs and reagents

SPRC was synthesized by the reaction of l-cysteine with propargyl bromide and then purified by recrystallization from an ethanol–water mixture (99%) as described previously.^21, 50^ Doxorubicin hydrochloride was purchased from Tocris Bioscience (Minneapolis, MN, USA). SC144 was obtained from Selleck Chemicals (Houston, TX, USA). WP1066 was purchased from Calbiochem (Billerica, MA, USA). LIF was acquired from Peprotech (Rocky Hill, NJ, USA). Antibodies to phospho-gp130 (Ser782, sc-12978-R), gp130 (sc-656) and *β*-actin (sc-47778) were purchased from Santa Cruz Biotechnology (Santa Cruz, CA, USA). Antibodies to phospho-STAT3 (Tyr705, 9145), STAT3 (9139), JAK2 (3230), Bcl-2 (2870), Bcl-X_L_ (2762), Survivin (2808), Bax (2772), Caspase-3 (9662), Caspase-9 (9508) and cytochrome *c* (4272) were purchased from Cell Signaling Technology (Beverly, MA, USA). Antibodies to MCL-1 (ab32087), Cyclin D1 (ab134175), COX IV (ab140643) and SERCA2 (ab150435) were purchased from Abcam (Cambridge, UK). Antibodies to CuZnSOD (10269), MnSOD (24127), Lamin B1 (66095) and α-Tubulin (11224) were purchased from Proteintech (Rosemont, IL, USA). Antibody to GAPDH (MB001) was purchased from Bioworld (Nanjing, China). Horseradish peroxidase (HRP)-conjugated secondary antibodies were from Jackson Laboratories (West Grove, PA, USA).

### Cell culture and treatments

Rat embryonic ventricular myocardial H9c2 cells were obtained from American Type Culture Collection (Rockville, MD, USA). Cells were cultured in Dulbecco's modified Eagle's medium with 4.0 mM l-glutamine, 4.5 g/l glucose (Invitrogen, Carlsbad, CA, USA) supplemented with 1% sodium pyruvate, 1% penicillin/streptomycin and 10% fatal bovine serum (Gibco, Grand Island, NY, USA) at 37 °C in a humidified atmosphere with 5% CO_2_. To explore the protective effects of SPRC on Dox-induced cardiotoxicity, cells were pretreated with SPRC (30 *μ*M) for 30 min prior to Dox (1 *μ*M) treatment. To determine the mechanism underlying the roles of SPRC, cells were pretreated with SC144 (a novel gp130/STAT3 pathway inhibitor) or siRNA against gp130 (gp130 siRNAs) prior to SPRC treatment.

### Animals and experimental protocols

Male 8-week-old C57BL/6 mice were purchased from Sippr-BK Experimental Animal Center (Shanghai, China), and housed under pathogen-free conditions with a free access to food and water. Mice were randomly assigned to six groups (six mice per group): Control group, Dox-treated group, Dox+SPRC-treated group, SC144-treated group, Dox+SC144-treated group and Dox+SC144+SPRC-treated group. In each of the Dox-treated group, Dox at a single dose of 15 mg/kg was injected intraperitoneally (i.p.) into the mice. SC144 was dissolved in DMSO (200 mg/ml) and then diluted to 1 mg/ml in saline with 40% propylene glycol and 5% Tween-80 for i.p. administration (10 mg/kg/day). SPRC (30 mg/kg/day, i.p.) was freshly prepared and injected 2 h after SC144 treatment. SC144 or/and SPRC were administrated 2 weeks before Dox and continued 5 days after Dox. Control mice were received the same volume of sterile isotonic saline. All mice were killed under anesthesia, and hearts were immediately harvested. For histological evaluation, 4% formaldehyde-fixed heart tissue specimens were stained with H&E and examined for histopathological evidence of cardiomyopathy. All experimental procedures were performed in compliance with the Guide for the Care and Use of Laboratory Animals published by the US National Institutes of Health (NIH) and approved by the Animal Ethical Committee of Fudan University (2015-0023).

### Echocardiography

Mice were sedated using 1.5% isofluorane, and were placed in a shallow left lateral position on a heating pad. Two-dimensional images were obtained using a high-resolution ultrasound system (Vevo 770; VisualSonics Inc., Toronto, ON, Canada) equipped with a mechanical scan probe. FS (calculated from short-axis images) and EF (calculated from parasternal long axis images) were calculated using the Vevo Analysis software.

### Cell viability and LDH release assay

Cell viability was assessed by CCK-8 assay. Briefly, after H9c2 cells were seeded in 96-well plates and received appropriate treatments, 10 *μ*l CCK-8 (Dojindo Lab, Kumamoto, Japan) solution was added to each well at a 1/10 dilution, followed by a further 1 h incubation. Absorbance was measured at 450 nm with a microplate reader (Tecan, Männedorf, Switzerland). The mean optical density of four wells in different groups was used to calculate the percentage of cell viability. LDH, a marker of cell damage or death, was evaluated using an assay kit (Beyotime Institute of Biotechnology, Shanghai, China) according to the manufacturer's instructions. In brief, LDH reduces nicotinamide adenine dinucleotide, which then converts a tetrazolium dye to a soluble, colored formazan derivative measured using a microplate reader (Tecan) at 490 nm.

### Co-immunoprecipitation

Cells were lysed in IP lysis buffer (20 mM Tris-HCl, 150 mM NaCl, 1% Triton X-100, pH 7.5) and incubated with gp130/STAT3 antibody and Protein A/G Plus- Agarose (Santa Cruz Biotechnology) according to the manufacturer's instructions. Normal IgG (Santa Cruz Biotechnology) was used as a negative control. Finally, immunoprecipitates were washed four times before boiling with 4 × loading buffer.

### Protein extraction and western blot analysis

NuPAGE 1 × LDS Sample Buffer (Invitrogen) was used to extract the total proteins of cells. NE-PER Nuclear and Cytoplasmic Extraction Reagents (Thermo Scientific, Waltham, MA, USA) were used to extract nuclear and cytoplasmic proteins of cells; and Mitochondrial Isolation Kit (Thermo Scientific) was used to isolate mitochondria components according to the manufacturer's instructions. Proteins from heart tissues were extracted in RIPA buffer (1% Triton X-100, 150 mM NaCl, 5 mM EDTA and 10 mM Tris-HCl, pH 7.0) containing phosphatase and protease inhibitor cocktail (Thermo Scientific). The protein extracts were subjected to centrifugation at 12 000 *g* for 15 min and loaded onto sodium dodecyl sulfate- polyacrylamide gels and then transferred to nitrocellulose membranes (PALL). After blocking with 5% nonfat milk in TBS (Amresco, Solon, OH, USA) containing 0.1% Tween-20, the membranes were incubated with primary antibodies overnight at 4 °C, followed by soaking in HRP-conjugated secondary antibodies and detected by Immobilon Western Chemiluminescent HRP Substrate (Millipore, Billerica, MA, USA). Signals were quantified by densitometry using a Bio-Rad Image Laboratory system.

### RNA interference

Two sets of small interfering (si)RNA oligos for rat *gp130* gene were synthesized by GenePharma (Shanghai, China). Target sequence for sigp130-1 is: (sense strand: 5′GCGCCAAGUUUCUGGUAUATT; antisense strand: 5′UAUACCAGAAACUUGGCGCTT); and for sigp130-2 is: (sense strand: 5′GGCACAGAGU UGAUAGUAATT; antisense strand: 5′UUACUAUCAACUCUGUGCCTT). For RNA interference, cells in six-well plates were transfected with 100 nM negative control siRNA (Santa Cruz Biotechnology) or gp130 siRNA using RNAiMAX (Invitrogen) according to the manufacturer's instructions. The medium was replaced at 8 h post-transfection, and silencing efficiency was determined by western blot 48 h after transfection.

### Immunofluorescence

Cells were cultured in Lab-Tek Chamber Slide System (Thermo Scientific) and exposed to appropriate treatments. After fixed with 4% paraformaldehyde, permeabilized with 0.2% Triton X-100 and blocked by goat serum, cells were incubated with antibodies recognizing gp130/STAT3 overnight at 4 °C, followed by Alexa Fluor 594/488-conjugated secondary antibodies (Invitrogen) incubation for 2 h. The cells were then counterstained with DAPI and visualized by confocal microscopy (Zeiss LSM 710).

### Hoechst 33258 and TUNEL staining

Cells were rinsed twice in PBS and fixed in 4% paraformaldehyde for 20 min. After washing, cells were incubated with Hoechst 33258 staining solution (Beyotime Institute of Biotechnology) at room temperature for 5 min. The apoptotic cells were then observed under a fluorescent microscope (Zeiss Inc., Oberkochen, Germany). DNA fragmentation was visualized by the TUNEL method using the TUNEL Apo-Green Detection Kit (Biotool, Houston, TX, USA). Cells were fixed and permeabilized. After being washed, cells were incubated with TdT terminal transferase and FITC-12-dUTP, and then counterstained with DAPI. For heart sections, apoptotic cardiomyocytes were examined using the *In Situ* Death Detection Kit (Roche, Branchburg, NJ, USA), with myocytes counterstained by *α*-actinin antibody (Sigma-Aldrich, St. Louis, MO, USA) and observed using a Zeiss fluorescent microscope. TUNEL-positive nuclei were quantitated by counting 1000 random cardiomyocytes and calculated using the Image J software (NIH, Bethesda, MD, USA).

### Flow cytometric analysis

The assay was performed by using Annexin V-FITC apoptosis detection kit (BD Biosciences, Franklin Lakes, NJ, USA) according to the manufacturer's instructions. Briefly, cells were harvested, washed with PBS, suspended in Annexin V binding buffer (10 mM HEPES, 2.5 mM CaCl_2_, 140 mM NaCl) and stained with Annexin V-FITC. The number of apoptotic cells was determined by flow cytometry using a BD FACSCalibur flow cytometer.

### ROS assay

Intracellular ROS was measured using 2'7'-dichlorodihydrofluorescein diacetate (DCFH-DA) as a fluorescent probe (Sigma-Aldrich). DCFH-DA is a non-fluorescent analog of fluorescein which will emit fluorescence after being oxidized by intracellular ROS. The bright fluorescence from the highly fluorescent DCF indicates the concentration and distribution of ROS.^51^ Cells were loaded with DCFH-DA (10 *μ*M) for 30 min, followed by washing with PBS. DCF fluorescence was detected using a fluorescence spectrophotometer (Tecan) with an excitation of 485 nm and an emission of 520 nm, and the fluorescence image was visualized using a fluorescence microscope (Zeiss Inc.).

### Mitochondrial membrane potential (Δ*Ψ*m) assay

*ΔΨm* was determined by an assay kit with JC-1, according to the manufacturer's instructions (Beyotime Institute of Biotechnology). JC-1 stains the mitochondria in cells with a high *ΔΨm* by forming red fluorescence J-aggregates, whereas in cells with depolarized mitochondria, JC-1 is present as green fluorescent monomers.^31^ In this way, mitochondrial depolarization was determined by a decreased ratio of red-to-green fluorescence intensity by a fluorescence microscope (Zeiss Inc.).

### Determination of intracellular [Ca^2+^]_i_

The level of intracellular [Ca^2+^]_i_ was measured using fluo-3/AM (Dojindo Lab), a fluorescent Ca^2+^-indicator probe. Briefly, after appropriate treatments, cells were washed with HBSS and loaded with fluo-3/AM (5 *μ*M) for 30 min at 37 °C. Extracellular dye was removed by washing with HBSS, and additional 30 min was allowed to hydrolyze fluo-3/AM. The fluorescence intensity of the intracellular [Ca^2+^]_i_ was detected under a fluorescence microscope (Zeiss Inc.) at an wavelength of 480 nm excitation/525 nm emission, and quantified by flow cytometry (BD Biosciences).

### Statistical analysis

Data were presented as mean±standard deviation (S.D.). Statistical comparisons between multiple groups were performed using one-way ANOVA, followed by Dunnet's *post hoc* test. *P-*value of 0.05 or less was considered statistically significant.

## Abbreviations

Bax: Bcl-2 associated X protein
Bcl: B-cell lymphoma
Dox: doxorubicin
EF: ejection fraction
FS: fractional shortening
gp130: glycoprotein 130
H2S: hydrogen sulfide
JAK2: Janus kinase 2
LIF: leukemia inhibitory factor
MCL-1: myeloid leukemia cell differentiation protein-1
ROS: reactive oxygen species
SERCA2: sarcoplasmic/endoplasmic reticulum Ca^2+^ ATPase
SPRC: *S*-propargyl-cysteine
STAT3: signal transducer and activator of transcription 3
TUNEL: terminal deoxynucleotidyl transferase-mediated dUTP nick end labeling
*ΔΨ**m*: mitochondrial membrane potential
