# Supplementary figures and images for: Gp130-mediated STAT3 activation by S-propargyl-cysteine, an endogenous hydrogen sulfide initiator, prevents doxorubicin-induced cardiotoxicity
Source: Cell Death Dis. 2016 Aug 18;7(8):e2339–. doi: 10.1038/cddis.2016.209 (PMC5108313; doi:10.1038/cddis.2016.209)

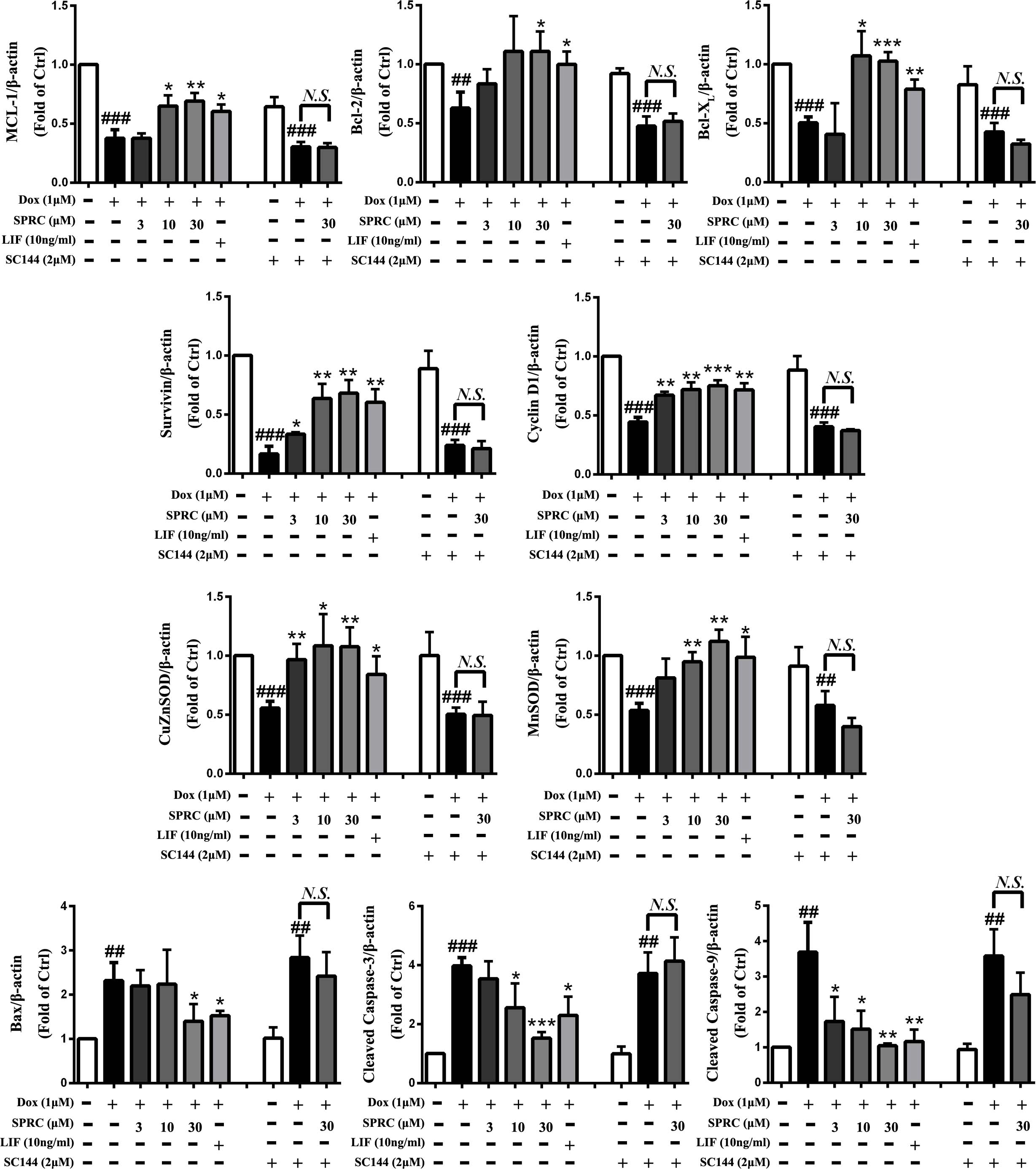

Supplement: Supplementary Figure 1 [file cddis2016209x1.tif]

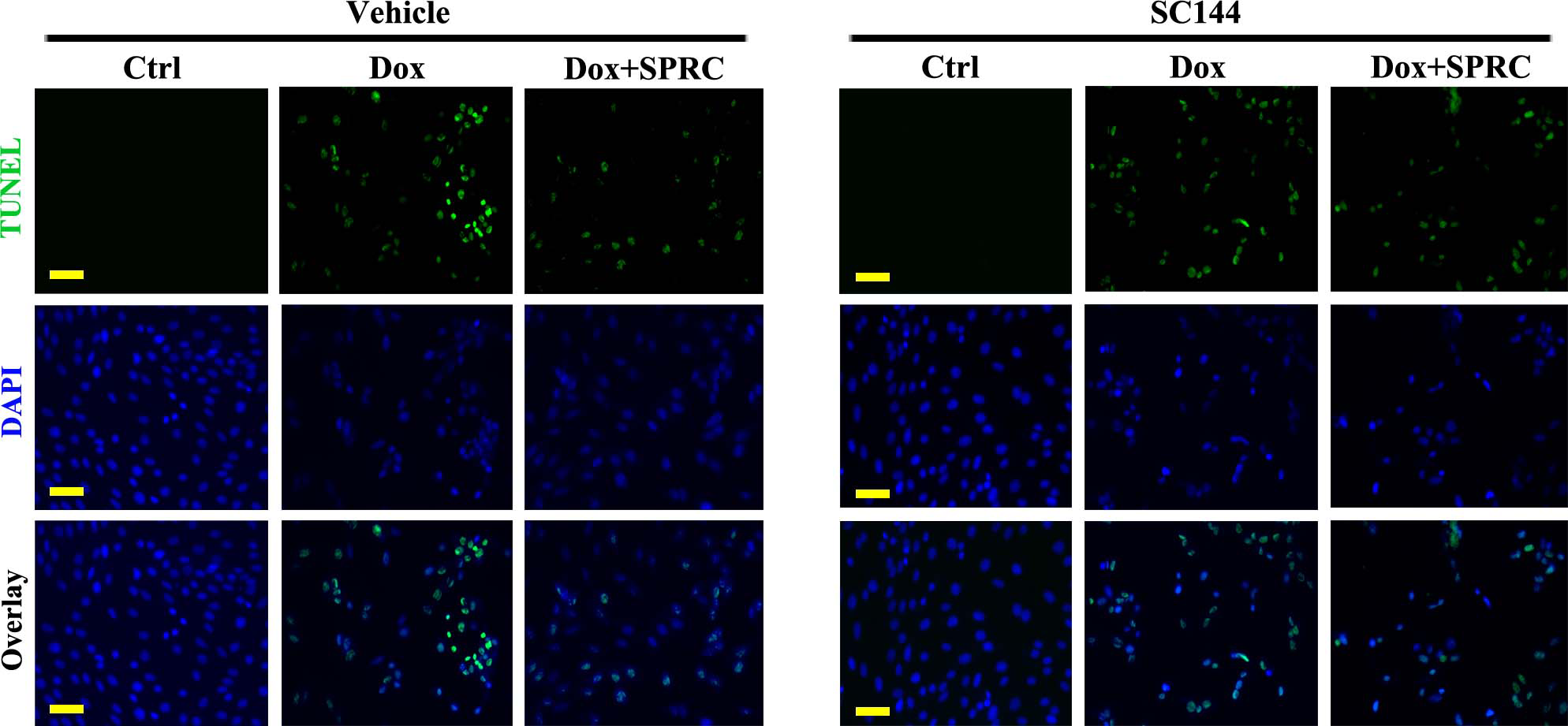

Supplement: Supplementary Figure 2 [file cddis2016209x2.tif]

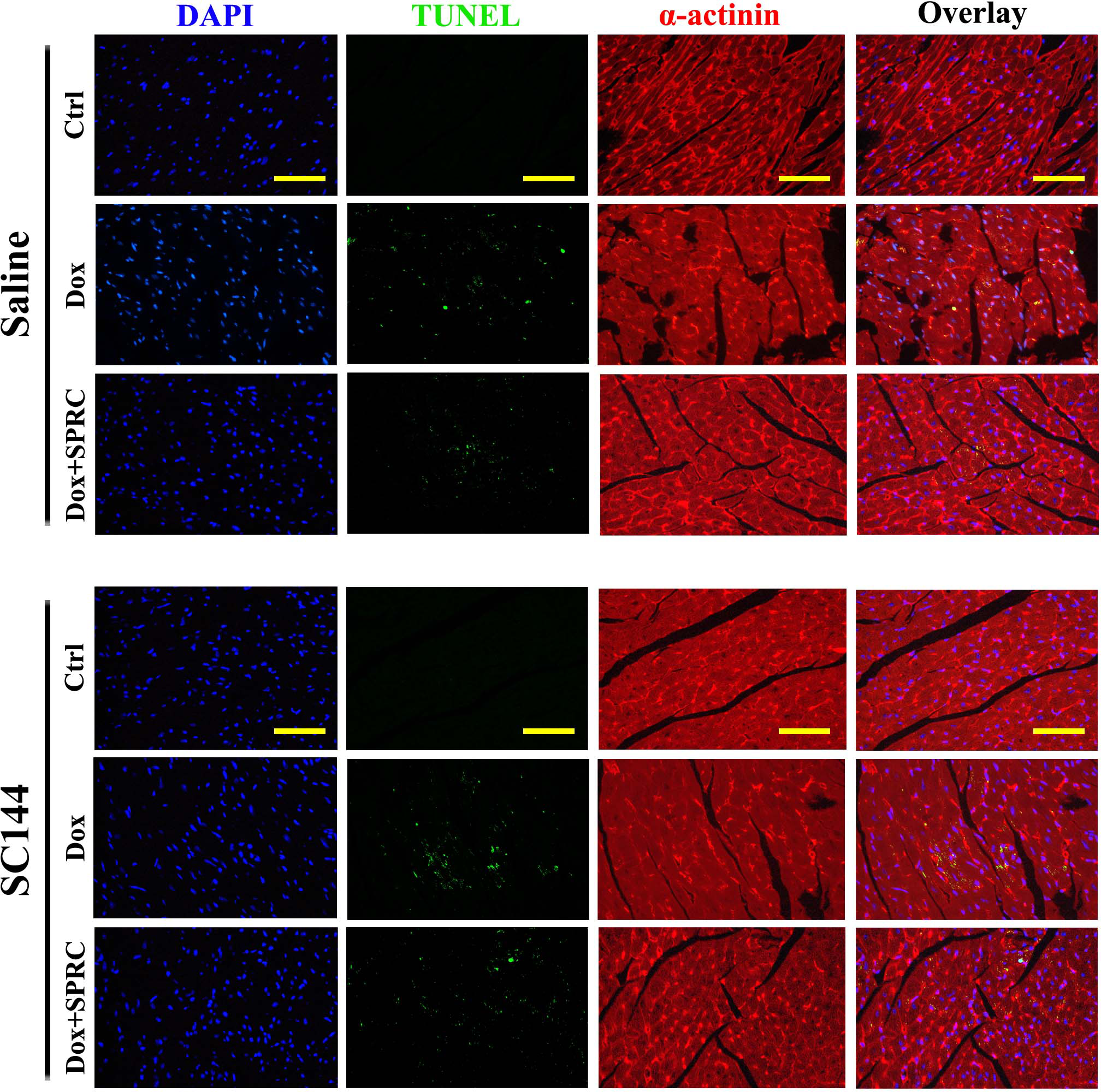

Supplement: Supplementary Figure 3 [file cddis2016209x3.tif]
